# A Retrospective Analysis of Atypical Cervical Cytology: Correlating Bethesda Categories with HPV Genotyping and Histological Follow-Up

**DOI:** 10.3390/jcm14238554

**Published:** 2025-12-02

**Authors:** Aleksandra Asaturova, Darya Dobrovolskaya, Andrew Zaretsky, Alina Badlaeva, Anna Tregubova, Aleksandra Rogozhina, Gennady Sukhikh

**Affiliations:** 1 National Medical Research Centre for Obstetrics, Gynecology and Perinatology Named After Academician V.I. Kulakov, Ministry of Health of the Russian Federation, 4 Oparina Street, 117513 Moscow, Russiaa-zaretsky@yandex.ru (A.Z.); alinamagnaeva03@gmail.com (A.B.); a_tregubova@oparina4.ru (A.T.);; 2 Department of Molecular Technologies, Research Institute of Translational Medicine, N. I. Pirogov Russian National Research Medical University, Ministry of Health of the Russian Federation, Bldg. 1, Ostrovityanova Street, 117997 Moscow, Russia

**Keywords:** cervical cancer screening, atypical squamous cells of undetermined significance (ASC-US), atypical glandular cells (AGC), human papillomavirus (HPV), HPV16 viral load, risk prediction, CIN2+, HPV genotyping

## Abstract

**Background/Objectives**: Atypical cytological findings in cervical screening, such as ASC-US, ASC-H, and AGC, present a clinical challenge due to their variable risk of underlying high-grade lesions. The precise stratification of this risk is crucial for effective patient management. This study aimed to correlate Bethesda cytology categories with HPV genotyping, including viral load, and histological follow-up to improve risk prediction for cervical intraepithelial neoplasia grade 2 or worse (CIN2+). **Materials and Methods**: In this retrospective single-center study, we analyzed 407 patients with cytological reports of ASC-US, ASC-H, or AGC. All patients underwent HPV DNA testing with genotyping for 21 types, with viral load quantification for HPV16/18, and subsequent histological verification. Statistical analyses included non-parametric tests, correlation analysis, and multivariate logistic regression to identify independent predictors of CIN2+. **Results**: The prevalence of CIN2+ differed significantly among the cytological categories: 23.2% in ASC-US, 47.3% in ASC-H, and 19.5% in AGC. ASC-H and a high HPV16 viral load were identified as independent predictors of CIN2+ in the multivariate analysis. An ASC-H result increased the probability of CIN2+ by 2.5 times (aOR = 2.51; 95% CI: 1.28–4.94). For each 1 log10 increase in HPV16 viral load, the risk of CIN2+ increased by 30% (aOR = 1.30; 95% CI: 1.16–1.46). Stratification of ASC-US cases by HPV16 status revealed a dramatically higher positive predictive value (PPV) for CIN2+ in HPV16-positive patients (66%) compared to HPV16-negative patients (12.6%). The AGC category showed the strongest association with glandular pathology, including adenocarcinoma in situ. **Conclusions:** The combination of cytological findings and HPV16 viral load provides a powerful model for risk stratification. An ASC-H result is a strong independent risk marker, while the clinical significance of ASC-US is fundamentally determined by HPV16 status. These findings advocate for a risk-based management algorithm that integrates liquid-based cytology with extended HPV genotyping and viral load assessments to optimize patient triage and follow-up.

## 1. Introduction

Cervical cancer is among the most common malignant diseases of the female reproductive system worldwide [1]. Each year, approximately 85% of cases occur in socially disadvantaged regions that lack organized screening programs and qualified personnel for standardized cytological diagnosis [2]. The incidence is particularly high in developing countries; for example, in some rural areas of China, the rate reaches 81 cases per 100,000 women, with the country accounting for about 14% of the global cervical cancer burden [3]. The disease progresses slowly, passing through stages of cervical intraepithelial neoplasia, which provides significant opportunities for early diagnosis and prevention. Modern screening methods include liquid-based cytology (LBC), which enables the detection of precancerous and cancerous processes, and testing for human papillomavirus (HPV). The clinical importance of HPV testing has become increasingly prominent, evolving into a primary screening method in many international guidelines. Furthermore, emerging molecular techniques such as mRNA testing for E6/E7 oncogene expression and DNA methylation assays of host genes are currently being investigated as promising biomarkers for improving the specificity of cervical cancer screening and triage strategies. This paradigm shift is grounded in robust evidence demonstrating that persistent infection with high-risk HPV (hrHPV) genotypes (16, 18, 31, 33, etc.) is a necessary cause in the majority of cervical cancer cases [4]. The oncogenic potential of hrHPV types is primarily mediated by the viral oncoproteins E6 and E7, which drive cellular transformation by degrading host tumor suppressor proteins p53 and pRb, respectively, thereby disrupting critical cell cycle checkpoints and promoting uncontrolled proliferation [1]. The integration of HPV testing into clinical practice is driven by its key advantages, particularly its higher sensitivity for detecting precancerous lesions (CIN2+) compared to cytology alone and its high negative predictive value, which allows for the safe extension of screening intervals. Consequently, while LBC remains a valuable tool for the morphological assessment of cellular abnormalities, HPV testing now provides the foundation for more reliable risk stratification, where the detection of HPV 16/18 warrants immediate colposcopic referral, while the presence of other hrHPV genotypes guides tailored management strategies, thus forming the basis for personalized patient management [4].

The Bethesda System (TBS) is used to standardize the interpretation of cytological reports, covering a wide range of pathologies—from atypical squamous cells of undetermined significance (ASC-US) and those suspicious for high-grade squamous intraepithelial lesion (ASC-H) to atypical glandular cells (AGC). Each of these categories carries different prognostic weight and clinical significance, necessitating an individualized approach to patient management [5].

Particular attention is given to the interpretation of AGC, as this category remains one of the most challenging and diagnostically ambiguous. AGC are rarely detected, which is associated with difficulties in sampling material from the endocervical canal and endometrium, and their morphological features often do not allow confident classification of the changes as inflammatory, reactive, or neoplastic processes. Within the TBS framework, AGC are subdivided by their presumed origin (endocervical or endometrial), as the management strategy and clinical significance of these findings differ substantially. At the same time, the literature indicates that 9–35% of patients with AGC are found to have precancerous or malignant lesions, including adenocarcinoma in situ (AIS) and invasive tumors [1,6].

Thus, the integration of cytological screening, HPV testing, and histological follow-up appears critically important for risk assessment and the development of diagnostic algorithms. In the Russian Federation, cervical cancer screening is primarily opportunistic, and while a national HPV vaccination program has been approved, its population-wide implementation remains in the early stages. However, in a number of regions, particularly those that are economically disadvantaged, such retrospective studies combining atypical cytological reports according to TBS, HPV genotyping, and biopsy results remain insufficient. This underscores the relevance of investigating the distribution of HPV genotypes, the frequency of various atypical findings, and their clinical-pathological significance for establishing quality standards and improving the effectiveness of screening programs, which constituted the aim of our study.

## 2. Materials and Methods

**Figure 1:**
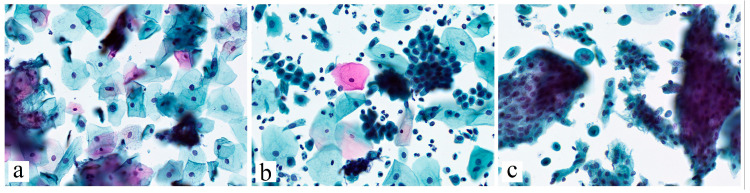
Main cytological categories included to the investigation (according to Bethesda system (2014)). (**a**)—ASC-US. The squamous cells with slightly hyperchromatic and enlarged nuclei, as well as irregular nuclear membrane contours; (**b**)—ASC-H. Groups of metaplastic cells with high N:C ratio, irregular nuclear membranes, enlarged and hyperchromatic nuclei; (**c**)—AGC. Clusters of glandular cells with nuclear enlargement, crowding and disordered arrangement. Papanicolaou stain, ×400.

This retrospective single-center study was conducted at the National Medical Research Center for Obstetrics, Gynecology and Perinatology named after Academician V.I. Kulakov of the Ministry of Health of Russia. The study analyzed a sample of 935 female patients under observation between 2019 and 2025 with cytological reports of ASC-US, ASC-H, or AGC. According to the inclusion and exclusion criteria, 407 patients were ultimately included in the analysis (Figure 1).

The inclusion criteria were as follows: (1) a cytological report of ASC-US, ASC-H, or AGC according to the Bethesda System (2014), (2) availability of qualitative and quantitative testing for high-risk human papillomavirus (HPV) DNA with genotyping, and (3) availability of histologically verified diagnosis obtained through colposcopy-directed biopsy or excisional procedure performed within 6 months of the initial cytological examination. These criteria were applied to select 407 patients from the initial screening population who had complete datasets for all three diagnostic modalities. The exclusion criteria were an incomplete set of diagnostic data and age under 18 years. Due to the retrospective nature of the study, the requirement for obtaining informed consent from patients was waived.

All patients underwent cytological examination of cervical smears, performed either by the conventional method or by liquid-based cytology (SurePath^®^; Becton Dickinson, Franklin Lakes, NJ, USA). Molecular biological testing for the detection of high-risk HPV DNA was conducted using real-time polymerase chain reaction (PCR) with type-specific detection for 21 HPV types (6, 11, 44, 16, 18, 26, 31, 33, 35, 39, 45, 51, 52, 53, 56, 58, 59, 66, 68, 73, 82) (DNA-Technology LLC, Moscow, Russia). Viral load quantification for HPV types 16 and 18 was determined through a standardized quantitative PCR protocol using calibration curves generated from serial dilutions of plasmid controls containing known copy numbers of the target HPV genes. The quantification cycle (Cq) values obtained from clinical samples were plotted against the standard curve to calculate the exact viral load expressed as log10 genomic equivalents per 10,000 cells. The analytical sensitivity of the assay was established at 3 × 10^3^ copies per mL, with a dynamic range of 3 × 10^3^ to 3 × 10^8^ copies per mL. Internal control targets were simultaneously amplified to monitor DNA extraction quality and PCR inhibition. Viral load values were categorized as low (<3.0 log), moderate (3.1–5.0 log), or high (>5.1 log) based on established clinical thresholds.

Histological verification of the diagnosis was performed through histological examination of cervical biopsy specimens stained with hematoxylin and eosin, following standard methodologies. To ensure diagnostic accuracy, all histological specimens underwent a thorough morphological evaluation by an experienced pathologist specializing in gynecological pathology. Histological reports were standardized according to the World Health Organization (WHO, 2020) classification.

Statistical analysis was performed using IBM SPSS Statistics 26.0 software (IBM Corp., Armonk, NY, USA). Quantitative data with a non-normal distribution (assessed by the Shapiro–Wilk test) were described using the median (Me) and interquartile range (IQR; Q1-Q3). Categorical variables were summarized as absolute and relative frequencies (n, %). The Mann–Whitney U test was used for comparisons between two independent groups on quantitative measures, while the Kruskal–Wallis test was applied for comparisons among three or more groups. Categorical variables were compared between groups using Pearson’s chi-square test or Fisher’s exact test, as appropriate for sample size. Associations between ordinal variables were assessed using Spearman’s rank correlation coefficient (ρ). The predictive value of cytological findings was evaluated by calculating odds ratios (OR) with corresponding 95% confidence intervals (CI). A *p*-value < 0.05 was considered statistically significant. Multivariate logistic regression analysis was performed to identify independent predictors of CIN2+, with HPV16 viral load analyzed as a continuous variable.

## 3. Results

The general characteristics of the study cohort are presented in Table 1.

As shown in Table 1, patients with ASC-US were significantly younger (median age 35.0 years) than those in the ASC-H and AGC groups (median age 37.0 years in both groups; *p* < 0.05). Analysis of the time intervals between cytological and histological examinations revealed statistically significant differences (*p* < 0.001). The observed right-skewed distribution of this parameter across all groups indicates that most patients underwent surgery within a relatively short timeframe; however, the presence of cases with significant delays justified the use of the median as a measure of central tendency. The shortest median interval was observed in the AGC group (21 days), followed by the ASC-H group (38 days), and the longest interval was in the ASC-US group (47 days). These findings may reflect differences in the perceived clinical significance of various cytological reports and, consequently, differences in the prioritization of invasive diagnostic procedures.

The presence and viral load of key high-risk HPV (hrHPV) genotypes were analyzed. The highest detection rate of HPV 16 was observed in the ASC-H group: 30.2% (39/129) of all ASC-H cases in the cohort and 50% (23/46) of cases with high viral load (>5.1 log). The proportion of HPV16-negative results in this group was the lowest (69.8%; 90/129) (*p* < 0.001). In contrast, the AGC group had the lowest proportion of HPV16-associated cases—7.3% (3/41). The ASC-US group had the highest absolute number of both HPV16-negative (80.2%; 190/237) and HPV16-positive (19.8%; 47/273) results, reflecting its heterogeneity (Table 2). The overall detection rate of HPV 18 was low (3.1%; 13/407), and no statistically significant association with cytological findings was observed (*p* > 0.05) (Table 2). Among other high-risk HPV genotypes (31, 33, 35, 39, 45, 51, 52, 53, 56, 58, 59, 66, 73, 82), statistically significant associations with cytological findings were found for HPV 56 (*p* = 0.018) and HPV 68 (*p* < 0.05) (Table 3). HPV 56 demonstrated a significant association with ASC-US findings, while the vast majority of HPV 68 infections (93.3%; 14/15) were diagnosed in the ASC-US group, with no cases detected in the ASC-H group (Table 3).

Analysis of histological reports revealed statistically significant differences in the frequency of high-grade lesions (CIN2+) between the groups (*p* < 0.001). The highest proportion of CIN2+ was observed in the ASC-H group—47.3% (61/129), comprising 57 cases of HSIL (CIN2-3), 1 case of adenocarcinoma in situ (AIS), and 3 cases of invasive squamous cell carcinoma (SCC).

In the ASC-US group, high-grade lesions were confirmed in 23.2% (55/237) of patients, with the most common outcomes being histological normality (39.7%) and low-grade squamous intraepithelial lesions (LSIL/CIN1—20.7%).

Although a normal histological picture was found in 51.2% (21/41) of cases in the AGC group, this group also had the highest proportion of adenocarcinoma in situ (4.9%; 2/41), underscoring its clinical significance for the diagnosis of glandular pathology (Table 4).

The results demonstrate significant differences in the frequency and structure of histologically confirmed lesions depending on the initial cytological finding. An ASC-H diagnosis has the highest predictive value for detecting CIN2+, whereas an AGC diagnosis, although less frequently associated with CIN2+, is crucial for identifying adenocarcinoma in situ.

Spearman’s correlation analysis revealed a statistically significant, weak positive correlation between the severity of histological and cytological findings (ρ = 0.111; *p* = 0.025). The strongest and most significant positive correlation was observed between the presence of HPV16 and the severity of histological changes (ρ = 0.306; *p* < 0.001).

To assess the predictive significance of the cytological findings, a risk analysis for detecting CIN2+ was performed. An ASC-H diagnosis was associated with a significant increase in the risk of CIN2+ (OR = 3.06; 95% CI: 2.00–4.68; *p* < 0.001). In contrast, the presence of ASC-US (OR = 0.44; 95% CI: 0.29–0.67; *p* < 0.001) and AGC (OR = 0.53; 95% CI: 0.24–1.15; *p* = 0.108) diagnoses was associated with a lower risk compared to other pathological findings (Table 5).

To evaluate the clinical significance of an ASC-US cytological finding, we performed a stratified analysis based on HPV16 status (Table 6). Among patients with ASC-US and a negative HPV16 test result (n = 190), the risk of CIN2+ was low (12.6%), while the negative predictive value (NPV) reached 87.4%. This indicates that a negative HPV16 test result in patients with ASC-US provides a high level of confidence for ruling out high-grade cervical lesions. In contrast, HPV16-positive patients (n = 47) demonstrated a substantially higher positive predictive value (PPV) for CIN2+ of 66%. This underscores the necessity of mandatory HPV testing (primarily for HPV16) to determine further management strategies for patients with this cytological finding.

To identify independent predictors of CIN2+ development, a multivariate logistic regression analysis was conducted. The model included the cytological findings ASC-US, AGC, and ASC-H; HPV16 viral load; and HPV16 monoinfection status (i.e., the presence of only this genotype).

The model was statistically significant (χ^2^ = 36.41; *p* < 0.001). As shown in Table 7, an ASC-H cytological finding and a high HPV16 viral load remained independent and statistically significant predictors of CIN2+. Specifically, ASC-H increased the probability of CIN2+ by 2.5 times (aOR = 2.51; 95% CI: 1.28–4.94; *p* = 0.008).

Furthermore, detection of a high viral load in patients with HPV16 demonstrated a strong dose–response relationship. For each 1 logarithmic unit (lg) increase in viral load, the risk of detecting CIN2+ increased significantly increased by 30% (aOR = 1.30 per 1 lg; 95% CI: 1.16–1.46; *p* < 0.001).

However, the presence of HPV16 alone (without co-infection with other types) was not an independent significant predictor (aOR = 1.07; *p* = 0.922), and the ASC-US cytological finding also showed no independent significant association with CIN2+ (aOR = 0.78; *p* = 0.642).

## 4. Discussion

This study provides important data for clinical practice, enhancing the understanding of the significance of ASC-US, ASC-H, and AGC cytological findings in relation to papillomavirus infection status. The results confirm existing data and reveal new patterns relevant for personalizing diagnostic and treatment approaches.

It is known that patient age significantly influences the overall risk of developing HSIL [7]. In our study, patients in the AGC and ASC-H groups were older (median age 37 years) compared to those in the ASC-US group (median age 35 years), consistent with the concept that long-term HPV persistence or epigenetic mutations increase the cumulative risk of more severe cervical lesions [7]. However, data on the influence of age on HSIL risk within each category remain ambiguous. For example, a study by Chen et al. showed that the risk of HSIL with ASC-H actually decreases with age—from 51.2% in women under 20 to 18.2% in patients over 51 [8]. Our multivariate analysis, which excluded age as a statistically non-significant predictor, confirms that age differences in the studied groups reflect the multifocal etiology of these cytological findings but are not an independent risk predictor within each group, necessitating the assessment of other, more significant factors.

The key role of HPV type 16 in the pathogenesis of squamous intraepithelial lesions of the cervix is also confirmed by our data [1,9]. Its high detection rate (30.2%) and the predominance of high viral load (50%) in the ASC-H patient group pathogenetically explain its aggressive potential. The results of the multivariate analysis quantitatively confirmed this relationship, revealing a strict dose–response effect: for each 1 logarithmic unit increase in HPV16 viral load, the risk of detecting CIN2+ increased significantly by 30% (aOR = 1.30; *p* < 0.001). This is associated with the ability of HPV16 oncoproteins E6 and E7 to induce genomic instability in the host cell, manifesting as HSIL cells [10]. Numerous studies demonstrate a clear relationship between HPV16 viral load and the risk of HSIL, cervical carcinoma in situ (CIS), and invasive squamous cell carcinoma, with even a low viral load increasing this risk by 10–20 times compared to HPV-negative cases [11,12]. Furthermore, data from several authors have shown that HPV16 is one of the main viruses with the highest load, and the risk of HSIL and more severe lesions increases with each logarithmic unit increase in its viral load [13,14,15]. An important clarification is that the presence of HPV16 alone (without co-infection with other types) was not an independent significant predictor in our model. This indicates that for risk stratification, the key factor is the detection and quantitative measurement of HPV16, not merely the identification of an isolated infection. Although the overall prevalence of HPV18 in our cohort was low, its role in glandular pathology should not be overlooked. Both cases of adenocarcinoma in situ (AIS) identified in the AGC group were associated with a high viral load of HPV18 (>5.1 log). This observation aligns with the known tropism of HPV18 for glandular epithelium and suggests that quantitative assessment of HPV18 may hold particular clinical value in the risk stratification of patients with glandular cell abnormalities, warranting further investigation in larger studies.

Conversely, the minimal association of the AGC cytological finding with HPV16 (3.4%) suggests that other factors play a leading role in the genesis of glandular lesions. This correlates with literature data, which indicate that the overall detection rate of hrHPV in AGC is low, approximately 21–30% [16,17]. This shifts the diagnostic focus towards non-HPV-associated mechanisms of carcinogenesis and necessitates the mandatory inclusion of methods aimed at detecting endometrial pathology in the examination algorithm [6,18,19]. It is important to note that in cases where AGC is associated with HPV, the risk of detecting adenocarcinoma in situ (AIS) and invasive cancer is high, especially when HPV16 (28.6%) or HPV18 (50%) is detected, compared to HPV-negative cases (10.4%) [16,20]. Meta-analyses have demonstrated that the presence of any hrHPV in AGC increases the probability of CIN2+ by 24.6 times, and HPV16/18 types by 49.5 times [17]. At the same time, a frequent histological findings among patients with an AGC cytological finding is histological normality (51.2%), reflecting the diagnostic difficulties in cytologically interpreting reactive changes in the glandular epithelium.

Given our results on the association of this finding with detected AIS cases, maximum oncological vigilance is fully justified, as confirmed by large studies in which AIS and invasive cancer were detected in 17.4–62.2% of cases [21,22].

Our data reveal a distinct distribution pattern for HPV68, characterized by its strong association with ASC-US findings (93.3% of all HPV68-positive cases) and a notable absence in the ASC-H category. This pattern suggests that HPV68 may possess a less aggressive pathogenetic profile compared to genotypes like HPV16, which is frequently detected in high-grade cytological abnormalities [23,24]. Consequently, the clinical significance of an ASC-US finding appears to be primarily determined by the presence of HPV16, while the implication of an isolated HPV68 detection may be different. The predominance of HPV68 and related alpha-7 group viruses in specific immunocompromised populations [25] further underscores the need to understand the host–pathogen interactions that shape its manifestation, warranting further investigation into its specific oncogenic risk.

Analysis of histological findings demonstrates the clinical significance of each cytological finding. The high frequency of HSIL/CIN2+ in ASC-H (47.3%) confirms its role as a marker of severe cervical intraepithelial lesions and is fully consistent with studies reporting detection rates ranging from 6 to 50.8% [26,27,28]. Multivariate analysis underscores the independent contribution of ASC-H to CIN2+ risk, increasing the probability of severe lesions by 2.5 times (aOR = 2.51; *p* = 0.008). This is pathogenetically justified, as the ASC-H finding reflects the presence of cellular atypia morphologically similar to HSIL, which is a direct consequence of active expression of viral E6/E7 oncogenes and integration of viral DNA into the host cell genome [10,13]. Thus, ASC-H can be considered an independent morphological marker of an aggressive course of HPV infection. However, according to our data, more than half of the cases (52.7%) showed less severe changes, indicating the heterogeneity of the ASC-H group and the need for thorough morphological evaluation to avoid overdiagnosis. This is consistent with the work of Gonzalez et al., where combined cytological features of ASC-H and LSIL were associated with a lower risk of CIN2+ detection (32.8%) [8,26].

The low positive predictive value (PPV) for detecting CIN2+ among patients with ASC-US (36.6%) confirms its “uncertain” status in patient management. However, stratification by HPV status significantly alters its clinical interpretation. Specifically, in the presence of HPV16, the predictive value of ASC-US increases to 66%, changing this finding from “uncertain” to a direct indicator of high risk that requires active diagnostic and treatment measures. This conclusion is strongly supported by the multivariate analysis, where the ASC-US finding lost statistical significance after adjusting for HPV16 viral load (aOR = 0.78; *p* = 0.642). Thus, the risk associated with ASC-US is almost entirely mediated by HPV16 viral load, and our data are fully consistent with current international risk-based management guidelines (ASCCP, 2019) [29,30]. Conversely, patients with ASC-US and a negative HPV16 test result showed a low risk of CIN2+ (12.6%), allowing for conservative management [29].

These data clearly demonstrate that the modern diagnostic approach should be based on an integrated assessment of clinical and laboratory findings, rather than on an isolated interpretation of the cytological result.

## 5. Conclusions

The data obtained highlight the need for a differentiated, risk-based approach to managing patients with atypical cytological findings. The combination of an ASC-H cytological result and quantitative assessments of HPV16 viral load provides the most informative model for stratifying the risk of CIN2+. In addition, the key role of AGC as a marker for the risk of glandular pathology is confirmed, necessitating thorough exclusion of AIS.

The most significant practical implication is the demonstration that the clinical significance of ASC-US is entirely determined by HPV16 status, providing a compelling rationale for the routine use of HPV testing as a decisive trigger for determining subsequent management strategy. The identified association between ASC-US and HPV68 requires further investigation to define the role of this genotype in risk stratification algorithms.

## Figures and Tables

**Table 1:** Baseline Characteristics and Diagnostic Intervals of Study Patients by Cytological Category.

Parameter	AGC (n = 41)	ASC-H (n = 129)	ASC-US (n = 237)	*p*-Value
**Age, median (Q1–Q3), years**	37 (31–45)	37 (30–47)	35 (29–42)	<0.05
**The interval between cytology and histology,** **median (Q1–Q3), days**	21 (14–35)	38 (21–60)	47 (28–75)	<0.001

Data are presented as median with interquartile range (Q1–Q3). *p*-values were calculated using the Kruskal–Wallis test for comparisons between the three cytological categories. AGC, atypical glandular cells; ASC-H, atypical squamous cells—cannot exclude HSIL; ASC-US, atypical squamous cells of undetermined significance.

**Table 2:** Association of cytological findings with presence and viral load of HPV types 16 and 18.

**HPV Type 16**					
HPV Status	AGC, n (%)	ASC-H, n (%)	ASC-US, n (%)	Total, n	*p*-value
HPV16−	38 (92.7%)	90 (69.8%)	190 (80.2%)	318	<0.001
HPV16+	3 (7.3%)	39 (30.2%)	47 (19.8%)	89	<0.001
Total, n	41	129	237	407	
**Viral load categories**					
HPV16 < 3.0 lg	1 (6.3%)	6 (37.5%)	9 (56.3%)	16	0.642
HPV16 3.1–5.0 lg	0	10 (37%)	17 (63%)	27	0.184
HPV16 > 5.1 lg	2 (4.3%)	23 (50%)	21 (45.7%)	46	0.005
**HPV Type 18**					
HPV Status	AGC n (%)	ASC-H n (%)	ASC-US n (%)	Total n	*p*-value
HPV18−	39 (95.1%)	124 (96.1%)	231 (97.5%)	394	0.478
HPV18+	2 (4.9%)	5 (3.9%)	6 (2.5%)	13	0.478
Total, n	41	129	237	407	
**Viral load categories**					
HPV18 < 3.0 lg	0 (0.0%)	1 (0.8%)	1 (0.4%)	2	0.728
HPV18 3.1–5.0 lg	0 (0.0%)	1 (0.8%)	2 (0.8%)	3	0.864
HPV18 > 5.1 lg	2 (4.9%)	3 (2.3%)	3 (1.3%)	8	0.312

Note: Data are presented as n (%), with percentages calculated within each cytological category (column percentages). Data were analyzed using the Chi-square test.

**Table 3:** Distribution of Patients by Cytological Finding and Status of Various High-Risk HPV Genotypes.

HPV Genotype	HPV Status	AGC, n (%)	ASC-H, n (%)	ASC-US, n (%)	*p*-Value
**HPV 31**	Negative	38 (92.7)	113 (87.6)	207 (87.3)	0.579
	Positive	3 (7.3)	16 (12.4)	30 (12.7)	
**HPV 33**	Negative	40 (97.6)	121 (93.8)	227 (95.8)	0.505
	Positive	1 (2.4)	8 (6.2)	10 (4.2)	
**HPV 35**	Negative	40 (97.6)	124 (96.1)	232 (97.9)	0.620
	Positive	1 (2.4)	5 (3.9)	5 (2.1)	
**HPV 39**	Negative	40 (97.6)	124 (96.1)	227 (95.8)	0.847
	Positive	1 (2.4)	5 (3.9)	10 (4.2)	
**HPV 45**	Negative	40 (97.6)	125 (96.9)	228 (96.2)	0.913
	Positive	1 (2.4)	4 (3.1)	9 (3.8)	
**HPV 51**	Negative	40 (97.6)	122 (94.6)	226 (95.4)	0.673
	Positive	1 (2.4)	7 (5.4)	11 (4.6)	
**HPV 52**	Negative	40 (97.6)	124 (96.1)	219 (92.4)	0.098
	Positive	1 (2.4)	5 (3.9)	18 (7.6)	
**HPV 53**	Negative	41 (100.0)	123 (95.3)	222 (93.7)	0.156
	Positive	0 (0.0)	6 (4.7)	15 (6.3)	
**HPV 56**	Negative	41 (100.0)	124 (96.1)	220 (92.8)	**0.018**
	Positive	0 (0.0)	5 (3.9)	17 (7.2)	
**HPV 58**	Negative	40 (97.6)	126 (97.7)	229 (96.6)	0.844
	Positive	1 (2.4)	3 (2.3)	8 (3.4)	
**HPV 59**	Negative	41 (100.0)	128 (99.2)	232 (97.9)	0.418
	Positive	0 (0.0)	1 (0.8)	5 (2.1)	
**HPV 66**	Negative	38 (92.7)	120 (93.0)	231 (97.5)	0.054
	Positive	3 (7.3)	9 (7.0)	6 (2.5)	
**HPV 68**	Negative	40 (97.6)	129 (100.0)	223 (94.1)	**0.002**
	Positive	1 (2.4)	0 (0.0)	14 (5.9)	
**HPV 73**	Negative	41 (100.0)	127 (98.4)	229 (96.6)	0.173
	Positive	0 (0.0)	2 (1.6)	8 (3.4)	
**HPV 82**	Negative	40 (97.6)	126 (97.7)	224 (94.5)	0.175
	Positive	1 (2.4)	3 (2.3)	13 (5.5)	

Note: HPV-human papilloma virus, TBS—Bethesda system. *p*-values were calculated using Fisher’s exact test due to small, expected cell frequencies. Bold values indicate statistically significant results (*p* < 0.05).

**Table 4:** Distribution of Histological Findings in Patients with Different Cytological Findings.

Histology (According to WHO 2020)	ASC-US (n = 237)	ASC-H (n = 129)	AGC (n = 41)	Total	*p*-Value
Normal epithelium	94 (39.7%)	29 (22.5%)	21 (51.2%)	144 (35.4%)	<0.001 *
Cervicitis	39 (16.5%)	23 (17.8%)	1 (2.4%)	63 (15.5%)	
LSIL (CIN1)	49 (20.7%)	16 (12.4%)	11 (26.8%)	76 (18.7%)	
HSIL (CIN2-3)	50 (21.1%)	57 (44.2%)	6 (14.6%)	113 (27.8%)	
AIS	2 (0.8%)	1 (0.8%)	2 (4.9%)	5 (1.2%)	
SCC	3 (1.3%)	3 (2.3%)	0	6 (1.5%)	
**Total**	41	129	237	407	

Note: Data are presented as n (%); AIS—adenocarcinoma in situ, SCC—invasive squamous cell carcinoma. *—Statistically significant differences in the distribution of histological findings between groups (χ^2^ test).

**Table 5:** Association of Cytological Findings with the Risk of CIN2+ Detection.

Cytology (TBS)	<CIN2+ n (%)	≥CIN2+ n (%)	Total, n	*p*-Value	OR (95% CI)
**ASC-H−**	215 (77.3)	63 (22.7)	278	<0.001	3.06 (2.00–4.68)
**ASC-H+**	68 (52.7)	61 (47.3)	129		
**AGC−**	250 (68.3)	116 (31.7)	366	0.108	0.53 (0.24–1.15)
**AGC+**	33 (80.5)	8 (19.5)	41		
**ASC-US+**	101 (59.4)	69 (40.6)	170	<0.001	0.44 (0.29–0.67)
**ASC-US−**	182 (76.8)	55 (23.2)	237		
**Total**	283 (69.5)	124 (30.5)	407		

Note: OR—odds ratio; CI—confidence interval. The reference group for OR calculation for each finding comprised patients with other pathological findings.

**Table 6:** Predictive Value of ASC-US Cytology for Detecting CIN2+ by HPV16 Status.

Parameter	Total (n = 237)	HPV16+ (n = 47)	HPV16− (n = 190)
**Histology CIN2+ (n)**	55	31	24
**Histology <CIN2+ (n)**	182	16	166
**Positive predictive value (PPV), %**	23.2	66	12.6
**Negative predictive value (NPV), %**	76.8	34	87.4

**Table 7:** Multivariate Analysis of Predictors for CIN2+.

Predictor	Adjusted OR (aOR)	95% CI	*p*-Value
**ASC-H**	**2.51**	**1.28–4.94**	**0.008**
**Viral load HPV16**	**1.30**	**1.16–1.46**	**<0.001**
ASC-US	0.78	0.28–2.19	0.642

Note: Bold values indicate statistically significant predictors (*p* < 0.05). The reference category for cytology was the absence of atypical changes. The estimate for AGC was statistically unstable and was excluded from the final interpretation. Age was excluded from the model as it was not a significant predictor. HPV16 viral load was analyzed as a continuous variable (per 1 log10 increase).

## Data Availability

The original contributions presented in this study are included in the article. Further inquiries can be directed to the corresponding author. Due to patient privacy and ethical restrictions, the raw data are not publicly available.

## Abbreviations

The following abbreviations are used in this manuscript:

ASC-H	Atypical squamous cell cannot exclude HSIL
ASC-US	Atypical squamous cells of undetermined significance
AIS	Adenocarcinoma in situ
AGCs	Atypical Glandular Cells
CI	Confidence interval
CIN2+	High-grade cervical intraepithelial neoplasia
HPV	Human papillomavirus
LBC	Liquid-based cytology
NPV	Negative predictive value
OR	Odds ratio
aOR	Adjusted Odds Ratio
PPV	Positive predictive value
Sn	Sensitivity
Sp	Specificity
TBS	The Bethesda System
